# Some Refractories Used in Dentistry

**Published:** 1916-02

**Authors:** Guy Stillman Millberry


### SOME REFRACTORIES USED IN DENTISTRY.

BY GUY STILLMAN MILLBERRY, D.D.S.

*Read before the Panama-Pacific Dental Congress, Section III., September* 2, 1915.

Dentists frequently have occasion to use refractory materials; indeed, they are using refractories of some sort in everyday practice, but have probably never considered them as such.

A refractory substance is one which is capable of withstanding high temperatures, without marked changes in form or composition. The value of refractories, in the industrial world, is in direct proportion to this, the predominating property.

All our silicate cements possess some of the properties of refractories, as do the porcelains, asbestos, slate, investment compounds, furnace linings, and many other substances which are a part of the equipment or supplies in a dental office.

Let us look into the origin of some of the more common refractories, then classify them and determine their value in dentistry.

Geologically speaking, rock includes all beds of sand, gravel, clay and volcanic ash, as well as granite, sandstone, and limestone. Rocks are classified according to their genesis, as:

1. Igneous rocks, which have solidified from molten material.
2. Sedimentary or stratified rocks, which are laid down chiefly under water by means of mechanical, chemical or organic agents.
3. Metamorphic rocks, those formed by subsequent alteration by wind or weather.

Most rocks consist of one or more minerals. Granite, for instance, though seemingly simple in substance, is a compound rock, while quartz and some of the calcium rocks are composed of but one substance. I will ask your indulgence for a moment while we consider these three classes somewhat in detail, because the refractories which we use originate from variable sources.

### IGNEOUS ROCKS.

Igneous rocks have been formed by the consolidation of molten material, usually forced upward from the depths of the earth. If it reaches the surface it is known as volcanic rock, and if not, as plutonic rock. Chemical analysis from all parts of the world show that the constituents are chiefly oxides of silicon, aluminum, iron, magnesium, calcium, sodium and potassium. When the oxide of silicon predominates they are acid in reaction, and when low in this oxide they are basic; in fact, silicates which constitute the largest part of the earth's crust are spoken of as salts of silicic acid.

The glassy rocks (and by glass is meant that group which solidify without crystallization, as obsidian), are of igneous origin, as are some minerals. They are usually massive and appear homogenous without evidence of stratification, foliation, or banding structures which are common to other forms of rock. Pumice, though metamorphic in appearance, is a glassy rock, its vesicularity being due to the rapid escape of water at high temperatures. Fossils are never found in true igneous rocks.

### SEDIMENTARY ROCKS.

Sedimentary rocks are of secondary origin, laid down as sediments on land or under water. They are derived from physical disintegration or chemical decomposition of pre-existing minerals, plants or animals, commonly known as weathering. When moved from their original source by water they are classed as aqueous sediments, such as shales and sandstones; if by wind, as æolian sediments as sand dunes, and if by glaciers as glacial sediments. They include all substances from silt to boulders, but the finer, more homogenous kinds are generally spoken of in these terms.

This sedimentary material is frequently bound together by the deposition of mineral matter, such as cuprous carbonate, and ferrous oxide, etc., which is held in solution by percolating waters, and it may be converted into solid rock by great pressure. Such rock is usually simple in composition, stable in character and may vary in thickness from a fraction of an inch to hundreds of feet.

### METAMORPHIC ROCKS.

Metamorphic rocks are deformations of pre-existing types, classed as mechanical when due to movements of the earth, or by wind, and chemical when altered by liquids, gasses or heat.

### ASBESTOS

Let us now consider some of these substances in detail. Asbestos is probably more commonly used than any of the others in laboratory practice. It is a mineral consisting of metasilicates of calcium and magnesium, with the latter smetimes replaced by iron, known as actinolite and a hydrous magnesium silicate known as serpentine, of which the chrysotile variety is the chief source of asbestos. This latter form is usually found in veins in massive serpentine. It may be of igneous or metamorphic origin, usually the latter, as an alteration product of magnesium silicate. Due to its fibrous flexible structure it may be woven into a cloth valuable for fire-proofing and insulating.

The decomposition of asbestos materials by acids varies, depending on the character of material and constituents, such as binders, etc. Pure asbestos as such is insoluble in acids, but when organic substances or inorganic compounds soluble in acids are used as binders, decomposition naturally follows.

I need not mention in detail the many uses of asbestos in fibrous or powdered form, but you are not familiar with a prepared product, marketed under various names as transite and asbestos lumber. It is a composition of asbestos and Portland cement formed under hydraulic pressure into slabs of varying dimensions, as 42x48 inches and 36x96 inches, with a thickness ranging from one-eighth inch to one inch. It is a very suitable material for laboratory bench tops where acids are not used, and especially to protect walls or woodwork from blast lamps and blow-pipe flames. The thicker slabs will withstand a reasonably heavy swagging force. Keasby Mattison Company and H. W. Johns Manville Company are manufacturers of this product.

### TALC SOAPSTONE

Talc, and soapstone, which is an impure form of talc, are very similar to asbestos chemically, the exception being that they possess more silicon and less water. In consequence of this they are somewhat different in physical appearance. Being more homogenous in appearance, somewhat amorphous in character, they are well suited for laboratory sinks where acids are being constantly used, and may be used for switchboards or as an insulating material. Durability, insolubility and good heat resisting qualities are characteristic properties of talc and soapstone.

### MICA

Mica is a very complex substance, an ortho-silicate of aluminum with one or more of the following elements combined with it, viz., potassium, lithium, magnesia or iron. It is very elastic and a non-conductor of heat and electricity, found usually in dikes. The darker colored micas containing the latter two elements are most infusible but more soluble in sulphuric acid. They possess a perfect basal cleavage, allowing them to be split into thin, tough, flexible plates, and as such are used in stove doors. Mica has been used as a door for electric furnaces, but at the temperatures necessary to fuse porcelain it frequently flakes off, falling into the furnace, and if the resistance wires happen to be exposed it may combine with them, thus aiding in their crystalization. Phlogopite or amber mica, a ferro magnesian variety, is used very extensively in commutators. It is the most satisfactory insulator and possesses about the same hardness as copper.

### CORUNDUM.

Corundum—aluminum oxide, a hard crystalline mass—is occasionally found in grains or shapeless masses. It is next to diamond in hardness, and, therefore, has been used extensively as a polishing grit or for grinding purposes. Magnetite corundum is known as emery.

Prior to the discovery of carborundum, which is a manufactured product, corundum was extensively used as various shaped polishing stones. Being non-cohesive, shellac was used as a binder. The heat generated by friction, unless water was used, reduced the stone to a plastic mass, which rendered it useless. Occasionally dentists make use of nitric acid to cleanse grinding and polishing stones of the accumulated debris and metal, and even though corundum is not soluble in acids, the binder is, and the procedure in so far as corundum points are concerned is a failure.

### SLATE

Slate is a metamorphic equivalent of muds and shales, but is not formed by sedimentation, as the latter are. It may be derived from aqueous sediment or from igneous origin, the latter form being exceptional. It represents the finest particles of mineral matter and its use and durability depends upon the texture and mineralogic constituents. The change is produced by dehydration and crystallization, though the mineral grains are too small to be seen by the naked eye.

Clay slates are formed by a uniform and gradual pressure upon mud beds, during which time the material is cemented together by carbonates of lime or magnesium, kaolin, or combinations of iron. Mica slates are formed by a metamorphic process in which the kaolin and feldspar have passed into mica, forming a crystalline fabric enclosing sedimentary particles. Nearly all slates originate in marine deposits of clay and sand.

The color ranges from black, attributed to carbonaceous matter, as marine organisms on the sea floor, through gray, green, red and purples. The corrugation is attributed to radiation of heat of earth and subsequent contraction. The constituent elements include many of the alkalies as well as the alkaline earth group, aluminum and iron with some carbon and phosphorus.

Slate is, of all refractories, the most suitable for bench tops, and it seems strange that manufacturers have not made use of it as such. From 20 per cent. to 40 per cent. of the quarried product is available for mill stock,. which includes all smooth forms for building purposes. Its value as a bench top is based upon the properties of insolubility and impermeability, especially with regards to acids, its high heat-resisting qualities, smoothness, durability, and non-liability to fracture under ordinary swagging force.

### CLAYS

Clay products are probably more commonly used and the clay industry more widely distributed than any other single mineral in the world. Every State and Territory in the Uniter States, except Alaska and Hawaii, produce burned clay products. In most of the clay working industries, especially the low grade products, as brick, tile, and pottery, the manufacturer is the miner as well, but with the high grade clay products this is not so. The chief properties of clay are: high shrinkage, variable plasticity, tensile strength, hardness, non-absorption, and color.

Clays are metamorphic rocks; that is, they are all derived from some other rocks, be it feldspar or limestone. They are classed as: (1) risidual, where the weathering out of some substances leaves a clay bank as a residue, or (2) transported where they are carried by water and deposited in banks.

Clay was probably primarily used for pottery, wateı carrying vessels and cooking utensils. Mention is made in an English record in 1744 of an application for a patent for making porcelain from a clay produced by the Cherokee nation of America. This incident does not, however, represent the earliest records of the clay industry, for besides of the ruins on the Mediterranean the Incas and Aztecs give evidence of having used clay. Prehistoric workings in the Appalachian Mountains present traces of crude implements in some of the dikes, and even though these dikes contained feldspar, mica and garnet, it is plausible to believe the search was for kaolin.

The clay we are interested in is the one chiefly used in porcelain, viz., kaolin with a formula of $Al_2O_3$ 2 $SiO_2 2H_3O$, and the substance from which it is derived, feldspar. Kaolinization is a process of decomposition and recombination in which potassium feldspar is converted into kaolin by a loss of $K_2O$ with some $SiO_2$, and an absorption of $H_2O$. In the beds the strata are seldom uniform, as the kaolin seems to filter down until it settles in a bed of approximately 98 per cent. kaolin, on quartz. The alteration process is simple one of the weathering, and the more plastic material is nearer the surface.

As previously stated, the silicates constitute the largest part of the earth's crust, and of this kaolinite, the hydrous aluminum silicate is the most common.

Reference is made by Felix Pascadis, M.D., Vice-President of the Chemical Society of Philadelphia, in an oration delivered in 1802, of the "infinite service . . . the various kinds of clay . . . possess from common brick to elegant works of porcelain."

The value which kaolin possesses is its plasticity and infusibility. Due to its high refractory value, it retains its form even though the particles may be completely enveloped in fluid feldspar. It also has a remarkable resistance to the solvent action of fluid feldspar.

The change in form of these refractory materials, which we might consider as fusibility, is known as the deformation point. It is a pyro-chemical process in which heat and time are factors. The same principle holds good in the fusion of porcelain. The deformation point is usually determined by seger cones, a clay product, graded to modify their form at indicated temperatures ranging from 1,150 degrees C to 1,850 degrees C at 20 degree intervals. Thermo-electric and optical pyrometers are unsatisfactory. Another method of testing is to obtain standard materials and make test mixtures. Kaolin is purified by a washing process in which sand wheels, mica troughs, screens, concentration tanks, filter presses and driers are used.

### FELDSPAR

Feldspars all include potassium, sodium and calcium, besides aluminum in silicate formation. Due to alteration reactions by weathering and the presence of water carrying $CO_2$ the alkalies are converted into soluble carbonates and are leeched out; the resultant compound being kaolin. Calcium silicates are likely to resolve into calcite. Feldspar in a porcelain mixture is the cementing mixture. When subjected to sufficient heat to merely soften it, it becomes a bond, but when fluid it acts as a solvent, producing a more homogenous mass. When feldspar is in excess it causes warpage. To avoid this, kaolin should constitute at least 50 per cent. of the mixture, a good formula being: feldspar 20, kaolin 50, and quartz 30, mixed with 50 grams of water. The fusing of porcelain is thus seen to be merely a physical process, though termed a pyrochemical one.

### QUARTZ

Theoretically, this is known as a pure crystalline silica, but it is rarely found pure. The best quality is known as sugar quartz, and is usually found in the vicinity of feldspar and kaolin dikes. One of the distinguishing physical features between quartz and feldspar is that the former does not possess cleavage. It is highly resistant to chemical decomposition, except in the instance of hydrofluoric acid, which really attacks all silicates, and it is infusible before the blow-pipe.

The so-called quartz glass possesses many properties which make it valuable to the dentist. When hot it can be plunged into cold water without danger of cracking. It is therefore, suitable as an annealing tray for gold, being superior to any of the forms of clay which in the course of time become roughened on the surface and partially decomposed.

In the form of tubes it may be wound with Nichrome wire for electric furnaces having a low temperature, though Nichrome nor any of the other resistant wires of the nickel chromium iron group have proven satisfactory in temperatures necessary for the fusion of porcelain. It could, however, be used satisfactorily for heating purposes, such as drying-out investments, etc., thus avoiding the use of the open flame.

### MAGNESITE

Magnesite or magnesium carbonate is a new type of refractory in so far as building construction is concerned. When calcined, pulverized and mixed with magnesium chloride, it makes a strong fireproof cement for partitions or floors. It is basic in character and, therefore, of value in treating basic ores in crucibles, but does not resist acids and is, therefore, unsuitable for general laboratory use.

In addition to these natural refractories, I wish to discuss two others, which, though native minerals or derived from them, are sufficiently modified by treatment to belong to manufactured products, and as such have been christened with trade names.

### ALUNDUM

Alundum, which is the mineral bauxite treated in an electric furnace until it gives practically pure fused alumina, is one of the best refractories for our use on the market to-day. It is crushed to grains of various sizes, then graded, refined and mixed with various binding materials.

When supplied in various shapes for various purposes, such as crucibles, filters, muffles, it is formed as desired and fired. The binding materials, unless they be of the nature of feldspar, are driven off by heat and a pure alumina remains. The melting point is 2,050 degrees C., or 300 degrees higher than platinum, and the latter substance can easily be fused in it. Its thermal conductivity is much higher than any of the other refractories, and consequently it is valuable as a lining for electric furnaces.

Aluminum oxidizes readily and does not give up its oxide freely. It therefore does not combine with platinum as does silicon or carbon, and there is less likelihood of crystallization of the latter metal. It is non-corrosive and has no chemical reaction, therefore is especially suitable for electric muffles.

### ZIRCONIA.

Zirconia is another trade product. In 1878, M. H. Klaproth, a German mineralogist, discovered that sixty-eight per cent. of the mineral zircon differed from the other substances in the native compound, a silicate, and on investigation it proved to be the oxide of an unknown element which was named zirconium. It occurs native in amorphous, crystalline and graphitic form, and is about as plentiful as carbon. It fuses at 2,350 degrees C. The natural oxide, eighty-four per cent, pure, is called zirconia.

When refined it is obtained as a pure white substance, and the M. P. is raised to 3,000 degrees C. It was first used to replace calcium oxide of the Drummond calcium light and later by Von Welsbach as a skeleton for Welsbach lights, though thorium is now used in the latter instance.

Possessing a low thermal conductivity, it is not as serviceable for electric muffles as alundum. Mixed with magnesia, phosphoric acid, glycerine or starch as a binder, it can be molded into form, dried for several days, and fired at from 2,000 degrees C. to 2,300 degrees C. When thus treated, it is impervious to liquids, strong acids and alkali fusion mixtures, and will withstand sudden changes in temperature.

In presenting this brief paper on refractories, my thought has been to bring before you some information which may be useful in some of the occasional problems which we have to contend with in dental practice.—*Dental Items of Interest.*

